# Pit and Fissure Sealant versus Fluoride Varnish for the Prevention of Dental Caries in School Children: A Systematic Review and Meta-Analysis

**DOI:** 10.1155/2022/8635254

**Published:** 2022-09-20

**Authors:** Tasneem Rashed, Nura Alkhalefa, Alaa Adam, Abdulaziz AlKheraif

**Affiliations:** ^1^Dental Biomaterials Research Chair, Dental Health Department, College of Applied Medical Sciences, King Saud University, Riyadh, Saudi Arabia; ^2^Alfaisal University, College of Medicine, Riyadh, Saudi Arabia; ^3^King Saud Medical City, Riyadh, Saudi Arabia

## Abstract

Children's dental health has become the main concern, due to the increase in caries prevalence amongst children. Pit and fissure sealant (PFS) and fluoride varnish (FV) are effective measures for preventing dental caries. However, the clinical efficacy of these interventions when compared to one another is uncertain. The aim of the present systematic review with meta-analysis was to compare pit and fissure sealants with fluoride varnish for caries prevention of first permanent molars among schoolchildren. This is a meta-Analysis, which involves randomized control trials that compare the effectiveness of PFS with FV within 24 months of follow-up. Five databases were searched from 1990 to 2019 to identify studies published in Arabic or English language. The risk ratio (RR) and 95% confidence intervals (CIs) were calculated using a random-effects model. A total number of 4 studies were included with overall of 1249 children in both groups. Three included trial reported caries increment of first permanent molars (FPM) with 24 months of follow-up, there was no statistical significance (RR: 0.65; 95% CI 0.31 to 1.35; *P* = 0.26 I2 = 89%). As regards DMFS increment, the analysis showed no statistical differences between FV and PFS in terms of lowering DMFS increment (MD: 0.09; 95% CI: −0.03 to 0.21). Findings of this meta-analysis proved there is no significant difference between PFS and FV in caries prevention efficacy of FPMs at 2 years' follow-up, emphasizing the use of FV since it is more affordable and easier to apply.

## 1. Introduction

The problems and costs of the burden of dental caries bedevil all countries around the world. A 2010 systematic analysis revealed that dental caries was the most prevalent disease among 291 medical conditions, affecting more than three billion people globally [1]. Tooth decay is known for its high costs [2]. With the high price of caries treatment, people tend to leave their teeth without treatment, resulting in severe pain, infection, and eventual loss of the affected tooth. Undoubtedly, losing teeth has a major impact on the quality of life by affecting individuals' nutrition, speaking, and aesthetic appearance [3].

Fortunately, there are several prevention methods that have been proven to be useful both in the prevention of caries and in arresting them, and there are several methods commonly used in dentistry. First, pit and fissure sealant is used to stop the development of bacteria that cause tooth decay in the fissures of posterior teeth. Glass ionomer cement (GIC) and resin-based sealants (RBSs) are the two most common forms [4]. The penetration of flowable composites can be influenced by different factors when they are used as fissure sealants. Heat and sonic vibration seem to significantly increase the penetration of the flowable composite when compared to the conventional method. Kim et al. revealed that the use of sonic vibration reduces the viscosity of flowable composite, hence improving penetration [5]. Second, fluoride varnish is a thin layer applied directly to the teeth. The three primary fluoride varnishes on the market are Bifluoride, Fluor Protector, and Duraphat [6]. Lastly, silver diamine fluoride (SDF) can be applied directly on carious lesions to slow the progression of caries or on caries-free surfaces to prevent caries. However, a single application of SDF is insufficient to arrest the progression of caries, and repetition is required [7]. Furthermore, SDF has been reported to be cost-effective when used as an adjunct to traditional restorative treatment [8].

More than two meta-analyses have been conducted comparing the effectiveness of PFS with FV in the prevention of dental caries [9–11]. However, there is no certainty as to whether PFS is superior to FV for caries prevention. This confusion is due to the conflict between the previous meta-analyses and the question of which randomized controlled trials (RCTs) should be included. Thus, it is essential to conduct a systematic review to look in depth at the included studies and to provide logical reasons for excluding studies. This systematic review and meta-analysis are intended to compare pit and fissure sealants with fluoride varnish for the prevention of caries in the first permanent molars of schoolchildren.

## 2. Methods

This manuscript was reported and recorded under the Preferred Reporting Items for Systematic Reviews and Meta-Analysis (PRISMA) statement guidelines [12]. This systematic review was registered with PROSPERO on 7 July 2022 (CRD42022146807).

### 2.1. Criteria for including Studies

The criteria for selection were defined according to the population, intervention, comparison, and outcome (PICO) study design schema.

### 2.2. Type of Studies

Randomized controlled trials with at least 24 months of follow-up were included. Split-mouth, quasirandomized trials, nonrandomized trials, and observational studies were excluded.

### 2.3. Participants

Participants were schoolchildren aged between six and 12 years who have a sound occlusal surface in the first permanent molars.

### 2.4. Intervention

This systematic review focused on studies that compared resin-based sealants with fluoride varnish. Articles that compared the glass ionomer sealant or glass ionomer cement with fluoride varnish were excluded.

### 2.5. Outcomes of Interest

The outcomes of interest were the incidence of dental caries on treated or untreated caries on each surface of the first permanent molars and changes in decayed, missing, and filled surface (DMFS) increments.

### 2.6. Search Strategy

The following electronic databases were searched: Embase, Google Scholar, the Cochrane Central Register of Controlled Trials (CENTRAL), and MEDLINE via Ovid for studies from January 1980 to May 2022. The full search strategy is included in Table 1 in the Supplementary Materials. For RCTs that are ongoing or completed but not yet published, ClinicalTrials.gov was used. The reference lists of identified articles were searched so that additional relevant studies could be identified.

### 2.7. Data Extraction and Quality Assessment

Two authors (TR and AA) screened the search results independently to extract the data utilizing Excel. The Cochrane Collaboration's tool was used to assess the risk of bias in the included study [13]. The articles were categorized as low risk, unclear, or high risk based on seven domains. When the reviewers disagreed, the third reviewer cast the deciding vote.

### 2.8. Statistical Methodology

The statistical analysis was carried out using Review Manager (RevMan) version 5.4. For the continuous outcome, the weighted mean difference was calculated with the corresponding 95% confidence interval (CI). When the outcome was dichotomous, the risk ratio (RR) with its 95% CI was calculated. Heterogeneity was measured using I^2^ statistics. When there was heterogeneity (*l*^2^ > 50%), a random-effects model was used.

## 3. Results

### 3.1. Search Results

**Figure 1:**
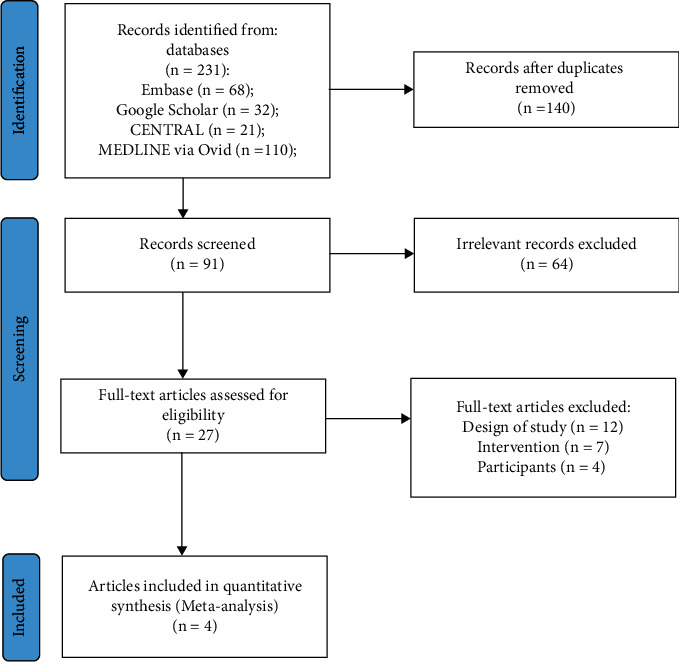
PRISMA flow diagram of included articles.

The initial search for studies yielded 231 candidates (Embase 68, Google Scholar 32, CENTRAL 21, and MEDLINE via Ovid 110; Figure 1). Of these, 140 duplicate studies were removed via EndNote reference management. After titles and abstracts were screened, 64 studies were excluded. The remaining 27 studies were assessed for eligibility, of which four RCTs met the eligibility criteria and were selected for meta-analysis [14–17].  Table 1 summarizes the characteristics of the studies. The four RCT studies were published from 1996 to 2014. The included studies involved 1249 subjects between the ages of six and eight.

### 3.2. Assessment of the Risk of Bias

**Figure 2:**
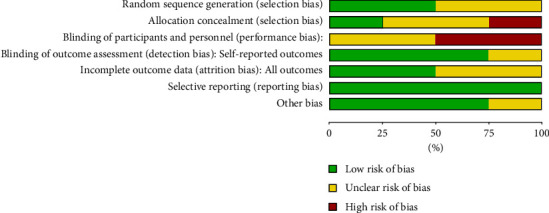
Risk of bias assessment of included studies.

The results of the risk of bias are illustrated in Figure 2. The selective reporting of the outcome in all studies was low risk. However, half of the studies were at high risk of bias because of the blinding of participants and personnel, and the other half were an unclear risk. One study illustrated methods of sequence generation, including computer-generated random numbers. Although Bravo et al. and Salem et al. mentioned that the subjects were selected randomly, the authors of the two studies did not mention the methods used for randomization [15, 17]. One study presented an unclear risk of detection bias. For attrition bias, one study had incomplete outcome data.

### 3.3. Meta-Analysis

**Figure 3:**
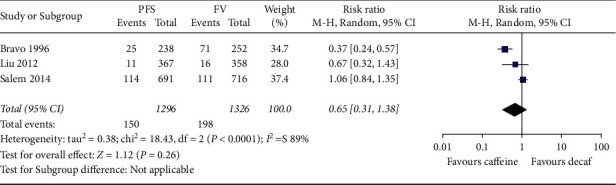
Meta-analysis of caries incidence of first permanent molars.

Three included trials reported the incidence of caries in first permanent molars (FPM), which included 2,622 FPMs. The follow-up time was 24 months among all included trials. Although the initial study showed that PFS had better efficacy than FV, the more recent study showed no difference in the comparison between both methods, indicating that there is no statistical significance in the meta-analysis (RR: 0.65; 95% CI 0.31 to 1.35; *P* = 0.26 *I*^2^ = 89%; Figure 3).

**Figure 4:**
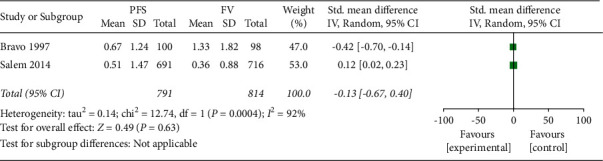
Meta-analysis of caries incidence DMFS (occlusal surfaces).

As for the secondary outcome, only two studies have provided data for the DMFS increment [15, 17]. Bravo et al. [15] fell in favour of PFS (MD: −0.64; 95% CI: −1.07 to −0.21). Salem reported that PFS illustrated higher occlusal caries increment (CI) than FV,^16^ but due to the high numbers of participants in Bravo et al.'s study [14], there was no statistical significance detected between PFS and FV (MD: 0.09; 95% CI: −0.03 to 0.21). The heterogeneity of this meta-analysis was very high (Chi^2^ = 11.69, I^2^ = 91%; Figure 4).

## 4. Discussion

The medical literature provides clear evidence of the burden of caries on all aspects of life, especially the direct impact on the quality of life [18]. Posterior teeth are the most susceptible to caries due to their morphology [9]. Since FPMs are the first to erupt among the posterior teeth, most caries occur in those teeth; thus, it is essential to put extra effort into preventing caries in FPMs. In dentistry, PFS and FV are methods that have been proven to be anticaries measures [19, 20]. However, there is debate on the relative clinical efficacy of both methods. This meta-analysis revealed that PFS is as effective as FV in preventing dental caries. The results of this study are consistent with those of Li et al., who reported that in six-to nine-year-old children using PFS or FV, they were not significantly associated with higher caries incidence or greater increase in DMFS at two to three years of follow-up [9]. It is worth mentioning that PFS is more challenging than FV to apply in a school setting, as this technique requires a steady child and proper tooth isolation. Nevertheless, applying FV requires less time and is more affordable.

Although the results of this review were consistent with Feifei et al., the included studies differed [9]. Feifei has included studies that have different follow-up times in one analysis, which is unreasonable, since the PFS loses its efficacy over time; also, Feifei incorporates studies comparing GIC with FV, although the retention rate of GIC is very low [9]. In almost all studies in which GIC or resin-modified glass ionomers were used as a pit and fissure sealant and applied on molars very soon after eruption, the retention rate was low [21–23]. A 2016 meta-analysis reported that PFS was more effective than FV in caries prevention [10]. Wright et al. [10] added studies in a reckless way without considering the inclusion and exclusion criteria, meaning that trials that compare PFS with water fluoridation were included [10]. Moreover, Wright compared a five-year study with a two-year study in one analysis without using subgroups [10]. Another meta-analysis favoured PFS over FV in preventing caries in FPM at a two-year follow-up [11]; however, an updated version of the study concluded that there is no certainty as to whether PFS is superior to FV or the other way around [24].

The effectiveness of pit and fissure sealant (PFS) varies according to the materials used. For instance, GIC has lower retention rates when used as a PFS than a resin-based fissure sealant [25]. Although most studies have reported that GIC has a low retention rate, glass ionomer has a cariostatic effect even after a complete loss. RBSs, on the other hand, have better retention rates [26, 27]. A research study was carried out at a dental clinic in the town of Raisio, Finland [25]. Fross et al. compared RBSs and glass ionomers to measure the retention rates of each material over the course of seven years [25] *(cite).* RBSs showed a significantly better retention rate of 82%. Fross also reported that the differences between the two materials in caries increment were small despite the dissimilarity in retention rate [25].

A recent meta-analysis was performed to assess the retention rate of GIC and RBSs and their effectiveness in preventing caries. Although the study concluded that there was no difference between the materials in terms of prevention, the retention rate of RBSs was much higher than GIC [28]. This may affect the efficacy of the GIC in the long run, which justifies excluding GIC from the present meta-analysis.

Retention depends on multiple factors, such as enamel conditioning, adequate isolation, material viscosity, and application techniques [29]. The efficacy of RBSs when used to seal fissures depends upon their retention. Enamel pretreatment can enhance the bonding of composite resin. For decades, air-abrasion has been used before bonding to remove the plaque and organic deposits and leave a clean tooth surface, hence improving material adhesion [30]. Scribante et al. [31] found that using erythritol as a pretreatment showed a promising improvement in failure rate in a split-mouth randomized clinical trial to investigate the bonding failure rates of orthodontic brackets [31]. The authors divided the children into two groups according to the pretreatment procedure and particle size. They reported that the group that received erythritol had a significantly lower failure rate than those who received sodium bicarbonate [31]. Perhaps using erythritol as a pretreating procedure could increase the adhesion values of resin-based fissure sealants. Thus far, no research has used erythritol as a pretreatment for PFS.

Comparatively, PFS and FV can play important roles in preventing molar incisor hypomineralization (MIH) progress. MIH is a condition where the enamel has a developmental defect. Molars and incisors are commonly affected by enamel hypomineralization patterns. In this condition, the enamel suffers from posteruptive breakdown and potentially dental caries and sensitivity due to a decrease in the enamel depth after the eruption, which exposes the dentin [32, 33]. Furthermore, as part of the prevention protocol and to prevent and reduce tooth sensitivity, patients should receive regular applications of FV [34]. If the enamel surface of the MIH molars is undamaged, RBSs with an adhesive application can be used to improve fissure sealant retention, therefore, preventing dental caries [34]. Until now, the causes of the disease have been unknown; however, a recent review looked at the pre-, peri-, and postnatal factors of the disease and suggested several factors, such as genetic and medical problems during pregnancy, making MIH a multifactorial disease, as reported in the review [35]. Consequently, MIH is unpredictable and cannot be controlled, and thus prevention is important.

In dental research, split-mouth design is common in studies. Its popularity comes from its ability to significantly reduce the predicted treatment effect's intersubject variability [36]. This means that each intervention is exposed to the same risk factors as the mouth is divided into two or more experimental parts randomly assigned to different interventions. However, the split-mouth design has a major downside, which is carryover: one intervention can contaminate the other intervention [37]. FV can affect the entire mouth, resulting in overestimating the effect of PFS. Therefore, spilt-mouth trials were excluded.

Certain limitations should be considered when interpreting the present meta-analysis results. First, despite strict inclusion and exclusion criteria, there was significant heterogeneity among the studies. Due to the high degree of variation between studies, the results must be interpreted with caution. The significant heterogeneity of the results could be due to the quality of the included studies. Second, most of the included studies did not adequately control for confounders, such as fluoride exposure, socioeconomic status, and parental educational level. Finally, since this meta-analysis included only four studies with two years of follow-up, the long-term efficacy of PFS and FV has not been established.

## 5. Conclusion

The findings of this meta-analysis proved there is no significant difference between the efficacy of PFS and that of FV in preventing caries in FPMs at two years' follow-up and emphasized the use of FV since it is more affordable and easier to apply. High-quality studies with longer follow-up periods are required. There is ample evidence that PFS and FV have protective effects. Randomized clinical trials addressing the long-term effects and trials focusing on specific populations, such as individuals from low- and middle-income nations, are needed to expand the generalizable applications.

## Figures and Tables

**Table 1:** Characteristics of included studies.

Author and year	Study period	Age group	Study design	Sample size	Sealant		Fluoride varnish		Sealant		Fluoride varnish	
					Caries	Total	Caries	Total	Mean (SD)	Total	Mean (SD)	Total
Bravo et al., 1996 [14]	24 month	6-8	RCT	362‬‬‬‬‬‬‬‬‬‬‬‬‬	25	238	71	252	—	—	—	—
Bravo et al., 1997 [15]	24 month	6-8	RCT	362	—	—	—	—	0.96 (1.24)	—	1.33 (1.82)	—
Liu et al., 2012 [16]	24 months	9.1	Randomized parallel	501	11	367	16	358	—	—	—	—
Salem et al., 2014 [17]	24 months	6-7	Randomized parallel	400	140	691	150	716	0.41 (0.92)	691	0.33 (0.77)	716

## Data Availability

All relevant data are within the manuscript.

## Supplementary Materials

Supplementary Materials Supplementary Materials include detailed information about each database's search strategy.
