# Supplementary material for: Pit and Fissure Sealant versus Fluoride Varnish for the Prevention of Dental Caries in School Children: A Systematic Review and Meta-Analysis
Source: Int J Clin Pract. 2022 Sep 20;2022:8635254. doi: 10.1155/2022/8635254 (PMC9553663; doi:10.1155/2022/8635254)
Supplement: Supplementary Materials — include detailed information about each database's search strategy. [file 8635254.f1.docx]

**supplementary file**

**Supplemental table 1: Search strategy for electronic databases**

*Search strategy for identification of studies*

Studies were identified by electronic searches of Embase, Google Scholar, CENTRAL, MEDLINE via Ovid.

**The following strategy was used to search**

**Scopus** [search conducted March 22, 2022]:

( children OR child OR student OR school OR schoolchildren ) AND ( Pit and Fissure Sealants OR Fissure sealant OR composite OR tetric) AND ( fluoride varnish OR fluoride OR varnish OR dental fluoride OR Fluorides, topical) AND ( randomizedcontrolled trial OR controlled clinical trial OR randomized OR clinical trial) ( LIMIT-TO ( LANGUAGE , "English" OR "Arabic" ) )

**ISI Web of Science** [search conducted March 22, 2022]:

#1 (ALL=( randomized OR controlled clinical trial OR randomizedcontrolled trial OR randomised OR clinical trial OR randomly OR trial ))

#2 (ALL=( Fissure sealant OR dental seal OR composite seal OR Resin cements OR Pit and Fissure Sealants OR Pit Sealants)) AND LANGUAGE: (English)

#3 (ALL=(fluoride OR varnish OR topical OR dental fluoride OR Fluorides OR fluoride varnish)) AND LANGUAGE: (English)

#4 (#1 AND #2 AND #3)

#5 (ALL=(Children OR Child OR student OR Primary teeth OR permanent teeth OR school OR schoolchildren)) AND LANGUAGE: (English)

5# #4 AND #5

**MEDLINE** via ovid search strategy [search conducted March 24, 2022]

#1 exp Child/
#2 student.mp.
#3 (school or children or school-children).mp.
#4 1 or 2 or 3
#5 exp Fissure sealan
#6  (dental seal or Resin cements or composite seal or Resin cements).mp.
#7  Resin seal $.mp.
#8  4 and 6 and 7
#9  exp fluoride/
#10 fluoride.mp.
#11 varnish $.mp.
#12  9 or 10 or 11
#13  8 and 12

**EMBASE** via Ovid search strategy [search conducted March 25, 2022]:

#1. child$.ti,ab.

#2. student$.ti,ab.

#3. (school$ or children$ or school-children$).ti,ab.

#4. Fissure sealant$.ti,ab.

#7. Varnish or fluoride or Resin or seal $.ti,ab.

#8. dental adj3 seal$.ti,ab.

#9. 4 and 6 and 7

**Saudi digital library** search strategy [search conducted April 1, 2022]

1 ALL=( randomized controlled trial OR controlled clinical trial OR randomized OR placebo OR clinical trials OR randomly)

2 ALL=( Pit and Fissure Sealants OR sealant OR composite OR sealant OR composite)

3 #2 AND #1

4 ALL=( fluoride OR fluor OR OR NAF OR Sodium fluoride OR phosphate fluoride OR Varnish

5 ALL=(Children OR Child OR student OR Primary teeth OR permanent teeth OR school OR schoolchildren

6 #5 AND #4

7 #6 AND #3

8 ALL=(preschool OR preschool children)

9 #7 NOT #8

10 (#9) AND LANGUAGE: (English) OR (Arabic)

Refined by: ORGANIZATIONS-ENHANCED: ( KING SAUD UNIVERSITY OR KING ABDULAZIZ UNIVERSITY OR IMAM ABDULRAHMAN BIN FAISAL UNIVERSITY OR KING SAUD BIN ABDULAZIZ UNIVERSITY FOR HEALTH SCIENCES OR TAIBAH UNIVERSITY )

**Google Scholar** search strategy [search conducted April 1, 2022]

“Children” or “schoolchildren” and “Fissure Sealants” or “Fissure” and “RCT” or “randomized” and “controlled trial” -malocclusion -gingivitis
